# Jacobsen syndrome associated with Shone’s complex: a case report

**DOI:** 10.1590/1984-0462/2025/43/2024136

**Published:** 2025-01-17

**Authors:** Andressa Brum, Larissa Valéria Laskoski, Fabiana Gonçalves de Oliveira Azevedo Matos, Luciana Paula Grégio d’Arce

**Affiliations:** a Universidade Estadual do Oeste do Paraná, Cascavel, PR, Brazil.

**Keywords:** 11q deletion syndrome, Craniofacial abnormalities, Congenital heart defect, Growth disorder, Síndrome da deleção 11q, Anormalidades craniofaciais, Defeito cardiaco congênito, Transtornos do crescimento

## Abstract

**Objective:**

The aim of this study was to report the case of a child with Jacobsen syndrome in order to provide phenotypic information about this rare genetic disorder.

**Case description:**

A 5-year-old female preschooler was diagnosed with Jacobsen syndrome by karyotype testing. She presented with a variety of craniofacial anomalies and malformations, including cardiac impairment, characterized by a cluster of malformations in the left ventricle in line with the diagnosis of Shone’s complex.

**Comments:**

Jacobsen syndrome occurs due to a deletion of contiguous genes on the long arm of chromosome 11 (11q). The main characteristics associated with this genetic disorder are short stature and delayed neuropsychomotor development, trigonocephaly and craniofacial dysmorphism, hematological alterations, and cardiac malformations, among others. Thus, the child is being monitored at a Craniofacial Anomalies Center with a multi-professional team in order to monitor her development and treat the associated comorbidities.

## RESUMO

**Objetivo:**

Relatar o caso de uma criança com síndrome de Jacobsen visando fornecer informações fenotípicas sobre esta desordem genética rara.

**Descrição do caso:**

Pré-escolar do sexo feminino, com cinco anos de idade, foi diagnosticada com síndrome de Jacobsen por meio do teste do cariótipo. Apresentou uma variedade de anomalias craniofaciais e malformações, incluindo comprometimento cardíaco, caracterizado por um conjunto de malformações no ventrículo esquerdo em consonância com o diagnóstico do complexo de Shone.

**Comentários:**

A síndrome de Jacobsen ocorre em razão de uma deleção de genes contíguos no braço longo do cromossomo 11 (11q). As principais características associadas a esta síndrome genética são baixa estatura, atraso do desenvolvimento neuropsicomotor, trigonocefalia e dismorfismo craniofacial, além de alterações hematológicas, malformações cardíacas, entre outras. Dessa forma, a criança está em acompanhamento em um Centro de Anomalias Craniofaciais com uma equipe multiprofissional a fim de acompanhar seu desenvolvimento e tratar as comorbidades associadas.

## INTRODUCTION

Partial deletion of chromosome 11q23.3-q25 in which contiguous genes are deleted, known as Jacobsen syndrome (JBS—OMIM: #147791), is a genetic abnormality with multiple dysmorphic features, first described in 1973 by Danish geneticist Petrea Jacobsen.^1,2^ Since its description, more than 200 cases of patients with JBS have been reported, with an estimated incidence of 1/100,000 births and a ratio of two females to one male (2:1).^3,4^

Some characteristics described for JBS are delayed growth and psychomotor development, trigonocephaly, characteristic craniofacial dysmorphism such as wide nasal bridge, short nose with anteverted nostrils, thin lips, retrognathia, dysmorphic ears with low implantation, bilateral camptodactyly, and hematological alterations such as thrombocytopenia or pancytopenia. There have been reports of patients with cardiac, renal, intestinal, and genital malformations, as well as malformations of the central nervous system and/or skeleton. The presence of ocular, auditory, and immunological alterations has also been described in the literature.^2,4^ The aim of this study was to report a case of JBS, a rare syndrome with clinical characteristics, which can resemble other cases, requiring a differential genetic diagnosis due to the nonspecific phenotypes of the syndrome and the limited information found in the literature.

## CASE REPORT

A 5-year-old female preschooler of Venezuelan origin, and the only child of non-consanguineous parents, has been under the care of a multi-professional team at a Craniofacial Anomalies Rehabilitation Center in the State of Paraná, Brazil since December 2022.

She was born at 36 weeks via cesarean delivery due to a heart disease diagnosed by ultrasonography. At birth, she weighed 1460 g and was 47 cm in height, which was classified as small for gestational age (SGA). The child was discharged from the hospital after two days, according to maternal reports collected during the first appointment. Additional information about the birth, such as Apgar score, head circumference (HC), chest circumference (HC), and abdominal circumference (AP) was not found in the documents provided by her mother.

During infancy, she exhibited mild neuropsychomotor developmental delay, sitting up between 8 and 9 months and walking at almost 2 years old. At the age of 4 years and 5 months, her parents sought medical care due to a suspicion of Turner’s syndrome.

**Figure 1:**
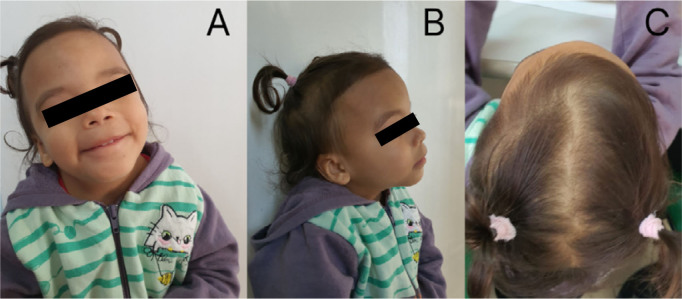
Images of the patient showing craniofacial malformations. Frontal view (A: ocular hypertelorism, telecanthus, ptosis, a downward-slanting palpebral fissure, a long filter, a wide nasal bridge, an anteverted nostril, thin lips, eyebrows that are far apart and slant downwards; right lateral view (B): low-set ears with a posterior turn; top view of the head (C): trigonocephaly.

Source: Authors.

**Figure 2:**
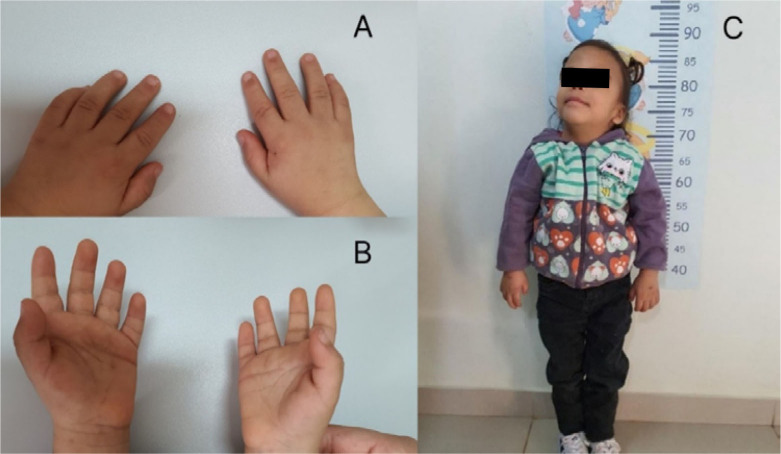
View of the hands and whole body, showing alterations in other regions of the body. Back of hands (A) and palm of hands (B): hands with short, tapered fingers; full body view (C): short neck and often adopts a posture with the head slightly reclined backward, possibly attributable to eyelid ptosis.

Source: Authors.

At present, the preschooler is 5 years old, weighing 10,100 g, and is 87 cm in height (both below the 3rd percentile, which has been evident from the fetal period until now). She has craniofacial alterations such as ocular hypertelorism, telecanthus, ptosis, downward-sloping palpebral fissure, long philtrum, wide nasal bridge, anteverted nostril, thin lips, low-set and downward-sloping eyebrows, low-set and posteriorly rotated ears, trigonocephaly, as well as alterations in other parts of the body such as a short neck, and short and tapered hands and fingers, and often adopts a posture with the head slightly reclined backward, possibly due to palpebral ptosis. Some of these features can be seen in Figures 1 and 2. In addition, she has a speech delay, being able to pronounce a few words, even with her mother, of the same foreign origin.

A Doppler echocardiogram indicated that the patient has Shone’s complex, a rare congenital disorder characterized by the presence of a series of obstructive malformations on the left side of the heart, of which the girl has the following alterations: coarctation of the aorta, bicuspid aortic valve, and moderate mitral stenosis. In addition, she also has mild left ventricular hypertrophy and mild ectasia of the ascending aorta.

Other tests were carried out to assess various systems, including a complete abdominal ultrasonography. However, no abnormalities were identified in the organs examined. In addition, the hematological tests carried out to investigate possible platelet alterations also showed results within normal parameters.

The proband presented karyotype 46,XX,del(11)(q23.3). However, her parents presented normal karyotype, characterizing the proband chromosomal alteration as de novo. Other genetic tests were not requested for the case due to the family’s choice.

## DISCUSSION

Jacobsen syndrome diagnosis can be made in both the postnatal period and the prenatal period, with the karyotype test.^5^ However, of more than 200 cases of JBS described in the literature to date, few have been diagnosed in the prenatal period, the oldest being in 1995 and the most recent in 2020.^6,7^

Turner’s syndrome was her pediatrician’s first diagnostic hypothesis and the reason because the proband and her family sought genetic counseling service. In fact, JBS shares some phenotypic characteristics with Turner’s syndrome and Noonan’s syndrome, such as short stature, a short and wide neck, and downward-sloping palpebral fissures.^5^

Recent research indicates that around 20% of patients diagnosed with JBS die within the first 2 years of life, in the vast majority of cases, either due to congenital heart disease or, in some exceptions, due to hemorrhage as a result of thrombocytopenia, and for the remaining 80% there is still no definite life expectancy.^5^ Although thrombocytopenia occurs in the vast majority of cases, the proband has not shown any platelet alterations so far. In the same way, some studies in the literature explain the absence of thrombocytopenia, in this syndrome, as a consequence of incomplete penetrance, despite the high incidence of 88.5%.^8-10^

Conversely, she presents a congenital heart defect, Shone’s complex. In 2004, they had already discovered a cardiac critical region of around 7 Mb on distal chromosome 11q, which supposedly contained a gene responsible for congenital heart disease in humans.^2^ Subsequently, other authors used chromosomal microarray mapping to study three patients with congenital heart defects and interstitial deletions on distal 11q, which coincided with the 7 Mb cardiac critical region identified previously. However, after testing, the authors concluded that the critical region for congenital heart defects is only 1.2 Mb, including the *ETS-1, FLI-1, KCNJ1, KCNJ5, P53AIP1*, and *RICS* genes, *ETS-1* being one of the genes expressed in rat embryos during early cardiac development.^11^

As for intellectual and psychomotor developmental delay, studies indicate that these are present in a mild or severe form in around 97% of cases, and there is also a correlation between the level of delay and the size of the deletion.^2,12^ Nevertheless, as previously reported, the family chose not to investigate the region by CGH array due to its high cost. The CGH array test would be important to assess the exact size of the deletion present in chromosome 11 of the preschooler, since the literature suggests that the larger the deletion, the greater the phenotypic consequences presented.^2^ Meanwhile, despite the patient’s mild motor and intellectual delay, she is able to attend school regularly due to follow-up with a multidisciplinary team, which has a significant impact on her motor and intellectual development.

Concerning her low weight and height for her age (both below the 3rd percentile), in 2004, researchers carried out a study of nine children diagnosed with Jacobsen’s syndrome, eight of whom had low height, only four had reduced levels of insulin-like growth factor-1 (IGF1), and the others had normal levels,^13^ as did the patient reported in this study, who also did not have a deficit in growth hormone (GH).

Therefore, it can be concluded that, although JBS has several associated clinical characteristics, the correlation between genotype and phenotype is still uncertain in this deletion, and cytogenetic examination is essential to confirm the case. Furthermore, the importance of multi-professional follow-up in cases of suspected and/or confirmed JBS is emphasized to provide a more accurate prognosis for the patient, since the condition can affect numerous systems, such as the cardiac, gastrointestinal, immune, renal, genital, as well as the nervous and/or skeletal systems.
